# Overexpression of wildtype EGFR is tumorigenic and denotes a therapeutic target in non-small cell lung cancer

**DOI:** 10.18632/oncotarget.6461

**Published:** 2015-12-04

**Authors:** Naiqing Xu, Wenfeng Fang, Libing Mu, Yanna Tang, Lei Gao, Shengxiang Ren, Dengfeng Cao, Lixin Zhou, Aiqun Zhang, Deruo Liu, Caicun Zhou, Kwok-Kin Wong, Lei Yu, Li Zhang, Liang Chen

**Affiliations:** ^1^ Graduate School of Peking Union Medical College, Beijing, China; ^2^ National Institute of Biological Sciences, Beijing, Beijing, China; ^3^ Chinese Academy of Medical Sciences, Beijing, China; ^4^ Collaborative Innovation Center for Cancer Medicine, Sun Yat-Sen University Cancer Center, Guangzhou, Guangdong, P. R. China; ^5^ Tsinghua University School of Medicine, Beijing, China; ^6^ Shanghai Pulmonary Hospital, Shanghai, China; ^7^ Cancer Hospital and Institute, Chinese Academy of Medical Sciences, Beijing, China; ^8^ Peking University Cancer Hospital, Beijing, China; ^9^ General Hospital of People's Liberation Army, Beijing, China; ^10^ Department of Thoracic Surgery, China-Japan Friendship Hospital, Beijing, China; ^11^ Department of Medical Oncology, Dana-Farber Cancer Institute, Boston, MA, USA; ^12^ Beijing Tongren Hospital, Capital Medical University, Beijing, China; ^13^ National Institute of Biological Sciences, Collaborative Innovation Center for Cancer Medicine, Beijing, China

**Keywords:** EGFR, transgenic mouse model, tyrosine kinase inhibitor, lung cancer

## Abstract

Current guidelines for lung cancer treatment with EGFR tyrosine kinase inhibitors (TKI) include only patients with mutated EGFR, although some patients with wildtype EGFR (wt-EGFR) have exhibited positive responses to this therapy as well. Biomarkers predicting the benefit from EGFR-TKIs treatment remain to be determined for patients with wild-type EGFR.

Here, we report that wt-EGFR overexpression transformed cells *in vitro* and induced tumorigenesis *in vivo* in transgenic mouse models. Wt-EGFR driven lung cancer was hypersensitive to TKI treatment in mouse model. Lung cancer patients with high-expression of wt-EGFR showed longer Overall Survival in comparison to low-expression patients after TKI treatment. Our data therefore suggest that treatment with EGFR inhibitors should be extended to include not only patients with mutated EGFR but also a subset of patients with overexpression of wt-EGFR.

## INTRODUCTION

Lung cancer is the leading cause of cancer-related deaths—approximately 1.38 million people died from lung cancer as of 2008 [1-3]. Chemotherapy remains the mainstay treatment in advanced lung cancer. Despite advances in multimodality therapies, however, the prognosis of lung cancer patients remains dismal.

Non-small cell lung cancer (NSCLC) is the major pathological type of lung cancer. More than 60% of NSCLC tumors express epidermal growth factor receptor (EGFR) [4]. EGFR is the founding member of ErbB family consisting of the four distinct receptors EGFR/erbB-1, HER2/erbB-2, HER3/erbB-3, and HER4/erbB-4. After ligand binding, these receptors homo- and hetero- dimerize and their tyrosine kinase domain is activated, initiating a cascade of events implicated in the development and progression of cancer through effects on cell cycle progression, apoptosis, angiogenesis, and metastasis. EGFR of this family is among the most intensely investigated kinase drug targets [5, 6].

21% of lung cancer patients are positive for somatic mutations in kinase domain of EGFR [7], although higher and lower mutational rates were reported. These mutations sensitize cancer cells to EGFR tyrosine kinase inhibitors (TKI). Remarkably rapid and often profound responses to gefitinib or erlotinib are frequently observed in a portion of lung cancer patients, in particular, non-smoker Asian females with pulmonary adenocarcinoma. Current guidelines provided by American Society of Clinical Oncology (ASCO) [8] and National Comprehensive Cancer Network (NCCN) (http://www.tri-kobe.org/nccn/guideline/lung/english/non_small.pdf) indicate that patient tumor samples must be positive for EGFR mutations to be eligible for TKI treatment.

Clinical studies have correlated EGFR mutational status with longer progression-free survival (PFS) after TKI therapy than conventional chemotherapy [9-16]. Interestingly, clinical data suggest that a small portion of patients with wt-EGFR respond to EGFR inhibitors. Patients negative for EGFR mutations have an objective response rate to TKIs of 1.1%-25.9% [9, 11, 17, 18]. Although the overall objective response rates are low, a few patients with wt-EGFR have had dramatic responses. Therefore, it is extremely important to identify the subset of patients with wt-EGFR who may respond to TKI treatment.

Early studies found that besides EGFR mutational status, the high EGFR copy number, higher EGFR expression or higher phospho-EGFR expression level was associated with higher response rates, longer time to progression and survival for total population [19-24]. However, for sub-population with wild type EGFR status, the predictive biomarkers for EGFR-TKIs remain unknown. Recently, *Wang et al.* reported that high EGFR copy number predicts benefits from tyrosine kinase inhibitor treatment for non-small cell lung cancer patients with wild-type EGFR [22]. This implies that some lung cancers may depend on wt-EGFR expression for maintenance. The critical remaining question is whether high expression of wt-EGFR is tumorigenic *in vivo* and whether tumors driven by wt-EGFR are sensitive to TKI treatment.

Here we show that a minor portion (9.8%) of lung cancer patients negative for EGFR mutations responded to TKI treatment. *In situ* tumor staining showed that EGFR expression was significantly stronger in responders than in non-responders. Importantly, we report for the first time on the development of lung cancer in a transgenic mouse model with lung epithelium-specific overexpression of human wt-EGFR and that these tumors are highly sensitive to TKI treatment. More importantly, NSCLC patients with overexpression of wt-EGFR showed longer overall survival (OS) after TKI treatment than patients with low expression of EGFR.

## RESULTS

### Patients harboring lung cancers overexpressing wt-EGFR respond to TKI

**Figure 1:**
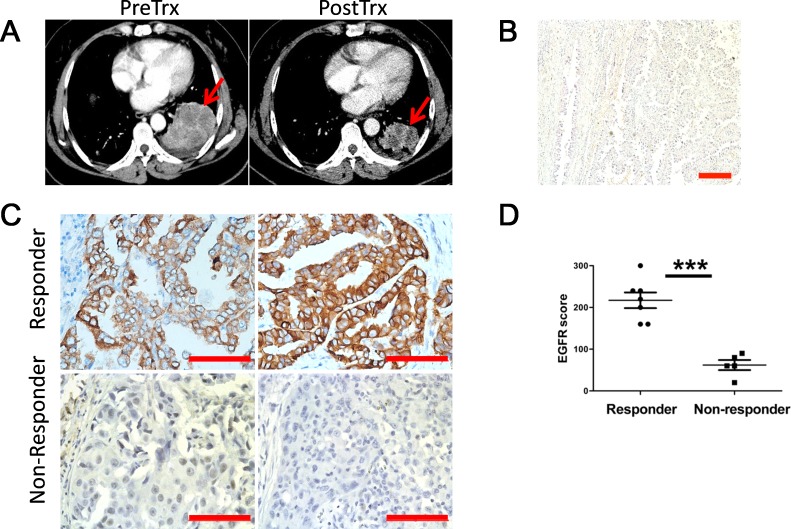
Lung cancer patients overexpressing wt-EGFR respond to TKI treatment **A**. Representative CT images for responders before and after Gefitinib treatment. Tumor is highlighted with read arrows. PreRx for before treatment; PostRx for after treatment. **B**. H&E examination revealed poorly differentiated lung adenocarcinoma pathological type in responders. **C**. IHC staining of representative responders and non-responders for EGFR expression level (scale bars, 200μm). **D**. Statistics of EGFR expression level comparison between responders and non-responders. Statistics was done on randomly picked 6 responders and 6 non-responders.

While administration of TKIs to EGFR mutation positive patients are well accepted in clinic, it remains controversial whether wt-EGFR patients should be treated with TKIs. Among the patients negative for kinase domain mutations in our clinic, we notice that around 9.8% of wt-EGFR patients showing partial regression of lung cancer, and another 52% stable disease. At the beginning of this study, we randomly collected tumor samples (provided by Drs. S.R. and C.Z. from Shanghai Pulmonary Hospital) from responders and non-responders with wt-EGFR. Tumor showed significant regression in responders in 5 weeks Gefitinib treatment (CT of a typical patient shown in Figure 1A). Tumor biopsy confirmed poorly differentiated lung adenocarcinoma pathology (Figure 1B).

Two possibilities could potentially explain drug sensitivity in these patients: 1) wt-EGFR overexpression is tumorigenic and thus sensitizes tumor cells to TKIs; or 2) tumors are driven by other kinases that can be coincidently inhibited by gefitinib. Earlier reports of kinome profiling showed that gefitinib is highly specific to EGFR [25, 26], suggesting that other kinases are unlikely to explain the sensitivity of these tumors to gefitinib. Therefore, we examined EGFR expression levels *in situ* with immunohistochemistry. Interestingly, we detected strong staining of EGFR expression in all of these 6 randomly picked patients (from S.R. and C.Z.) that respond to TKI, but low or no expression in all of 6 randomly picked non-responders (from S.R. and C.Z.) (Figure 1C). Moreover, the difference is significant (Figure 1D). Thus our data suggested that overexpression of wt-EGFR is tumorigenic and sensitizing tumor cells to TKI.

As these tumors were regarded as EGFR-mutation negative based on routine checking of L858R and exon 19 deletion in our clinic, possibilities of rare untypical mutations may contribute to TKI sensitivity. We therefore validated mutational status by checking thoroughly all the hot and rare mutations of EGFR as well as other candidate oncogenes through OncoCarta™ Panel v1.0. This assay enabled us to check 54 mutations at 44 sites of EGFR gene and 184 mutations in other 18 frequently mutated proto-oncogenes (sites listed in Supplementary Table 1). We randomly chose 6 samples of patients showing partial regression and 2 stable disease (provided by Drs. W.F. and L.Z. from Sun Yat-Sen University Cancer Center). Interestingly, we didn't detect any mutation in EGFR gene in all of these patients. However, we did detect KRAS G12C mutation in patient# 435771 and KIT L576P in patient# 430898 (Supplementary Table 2), both showing stable disease for over 2 years. Our data of 6 patients negative for mutations in all proto-oncogenes thus confirmed TKI sensitivity in a portion of EGFR mutation negative patients.

### EGFR overexpression activates downstream signaling and is transforming

**Figure 2:**
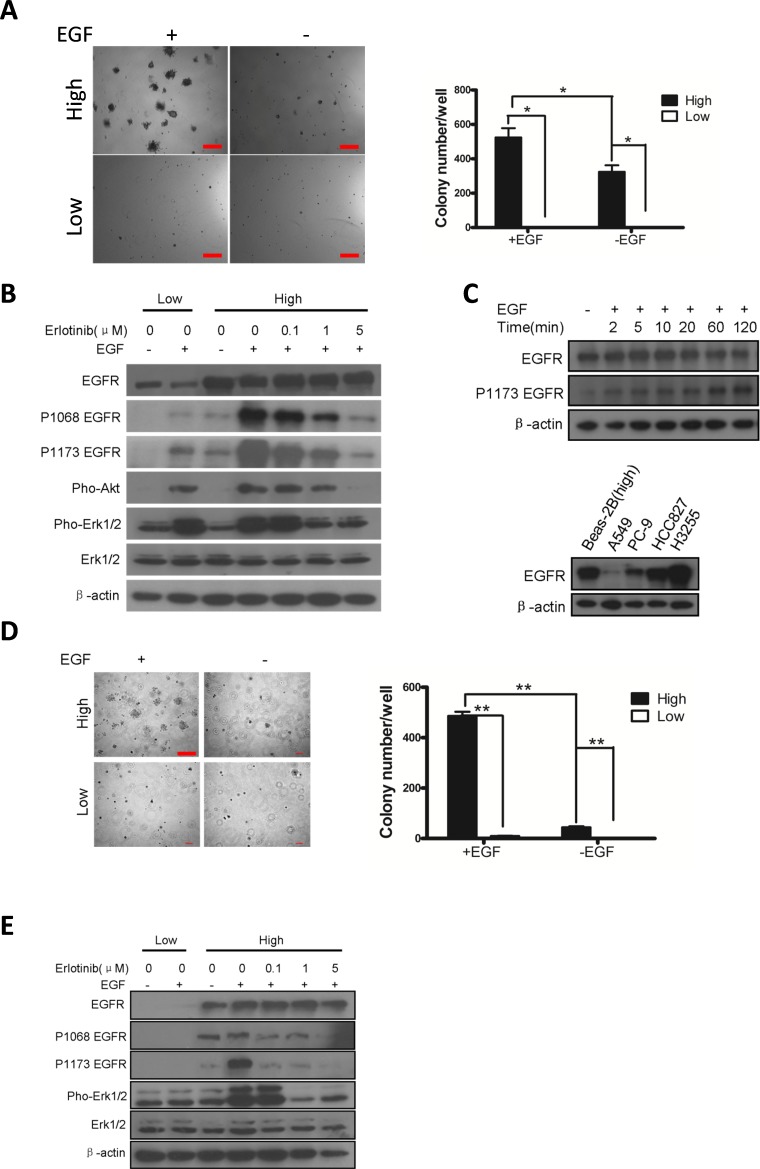
Overexpression of wt-EGFR is transforming **A**. Overexpression of wt-EGFR is transforming in Beas-2B cell line in presence of EGF by soft-agar assay. Left panel for typical pictures of soft-agar colony; Right panel for statistics of soft-agar colony. **B**. Overexpression of wt-EGFR activates downstream signaling. High- and low- wt-EGFR expressing Beas-2B cell line were subjected to western analysis to assess activation of EGFR, Akt, and Erk. Erlotinib is applied in high-expressing cells to check the ability to inhibit above mentioned signals. All the gels were run under the same experimental conditions. **C**. Overexpression of wt-EGFR maintains high protein level in cells. wt-EGFR overexpressing Beas-2B cells were stimulated with EGF for indicated time (minutes) and western blot analysis was done with antibody for total EGFR, p-Y1173 EGFR and actin (upper panel). Expression level of EGFR in Beas-2b overexpressing wt-EGFR were compared to widely used lung cancer cell lines (lower panel). **D**. Overexpression of wt-EGFR is transforming in NIH-3T3 cell line in presence of EGF. Left panel for typical pictures of soft-agar colony; Right panel for statistics of soft-agar colony. (scale bar, 400μm) **E**. Overexpression of wt-EGFR activates downstream signaling. High- and low- wt-EGFR expressing 3T3 cell line were subjected to western analysis to assess activation of EGFR, Akt, and Erk. Erlotinib is applied in high-expressing cells to check the ability to inhibit above mentioned signals. All the gels were run under the same experimental conditions.

Our clinical data that the wt-EGFR over-expressing tumors are sensitive to EGFR TKIs hint that wt-EGFR overexpression is tumorigenic. We next checked transforming ability of wt-EGFR overexpression on cell lines. We first expressed wt-EGFR in Beas-2B cell, a human lung bronchial epithelial cell lines. CAG promoter [27] is the strongest promoter characterized so far, enabling us to mimic the strong wt-EGFR expression in patients. Meanwhile, in our experience we have noticed that doxycycline inducible Tet-On system exhibits leakiness, such that low level expression of transgene can be detected in the absence of doxycycline administration. We took advantages of the leakiness to mimic the low expression of wt-EGFR in our experiment. The transfected cells were then subjected to soft-agar culture to verify the ability of survival under anoikis condition, a typical readout of transformed status. Interestingly, in the presence of EGF stimulation, wt-EGFR overexpressing cells forms significant more colonies than low expressing cells, which forms very few small ones (Figure 2A, left panel for typical microscopic photograph, and right panel for statistics).

Next we evaluated signaling events elicited by EGFR overexpression. It's been reported that phosphorylated EGFR goes through RAS, which activates PI3K-Akt and Raf-Mek-Erk pathways. As expected, we detected strong phosphorylated EGFR signal in high expressing, but not low expressing cells. We found phosphorylated EGFR, Akt, and Erk signals in high-expressing cells even without EGF stimulation(Figure 2B), consistent with an earlier report by Zhang *et.al.* [28], who showed that wt-EGFR was activated at high local concentrations. Activation of these signals was also sensitive to erlotinib inhibition, as we found that phospho-EGFR (P-EGFR), P-Erk, and P-Akt were sensitive to erlotinib treatment (Figure 2B).

One important aspect of lung cancer-related mutant EGFR is that the protein undergoes impaired C-cbl mediated degradation stimulated by EGF [29-31]. Since wt-EGFR overexpression is transforming, we next examined whether overexpressed wt-EGFR underwent degradation after EGF stimulation. We found that total EGFR was largely unaltered at the time checked (minutes after EGF stimulation): 0, 2, 5, 10, 20, 60, and 120. Phospho-EGFR was very low in cell not stimulated with EGF, and increased and remained at steady level from 2 minutes to 20 minutes after EGF stimulation. We saw a further increase in Phopho-EGFR level at 60 and 120 minutes after EGF stimulation (Figure 2C, upper panel). This suggested that overexpressed wt-EGFR mimicked mutant EGFRs seen in lung cancer patients, which are less responsive to EGF stimulation mediated downregulation.

We used CAG promoter to drive strong expression of wt-EGFR in Beas-2b cells. We asked whether this artificial cell line mimicked human lung cancer cell line sensitive to EGFR TKI treatment at EGFR expression level. We have compared EGFR expression of our Beas-2B against A549 (Kras mutant lung cancer cell line), PC-9 (EGFR exon19 deletion mutant lung cancer cell line), HCC827 (EGFR exon19 deletion mutant lung cancer cell line) and H3255 (EGFR L858R mutant lung cancer cell line). Result showed that our Beas2-CAG-EGFR cells express high amount of EGFR, equivalent to HCC827. It expresses lower amount of EGFR than H3255, but higher than PC-9, and much higher than A549 (Figure 2C, lower panel).

NIH-3T3 (hereafter 3T3) is a popular cell line for assessing transformation and expresses undetectable amounts of endogenous murine EGFR. Therefore, we evaluated the ability of wt-EGFR overexpression to transform 3T3 cells. We used CAG promoter to drive strong expression of wt-EGFR. As we failed to detect leaky expression of wt-EGFR in 3T3 cells using Tet-On system, we then used Rosa26 [32] promoter to drive low expression of wt-EGFR. In the presence of EGF, overexpression of wt-EGFR in 3T3 cells led to robust soft agar colony formation, in striking contrast to cells expressing low levels of EGFR (Figure 2D, left panel for typical microscopic photograph, and right panel for statistics).

We also assessed signaling events in these high- and low-EGFR expressing cells. Similar to Beas-2B background, we detected that EGFR and ERK are phosphorylated in cells with high expression of EGFR, but not in low expressing cells. Likwise, phosphorylation of these proteins are sensitive to erlotinib treatment (Figure 2E).

### wt-EGFR overexpression is tumorigenic in transgenic mice and tumor maintenance is dependent on continuous expression of EGFR

**Figure 3:**
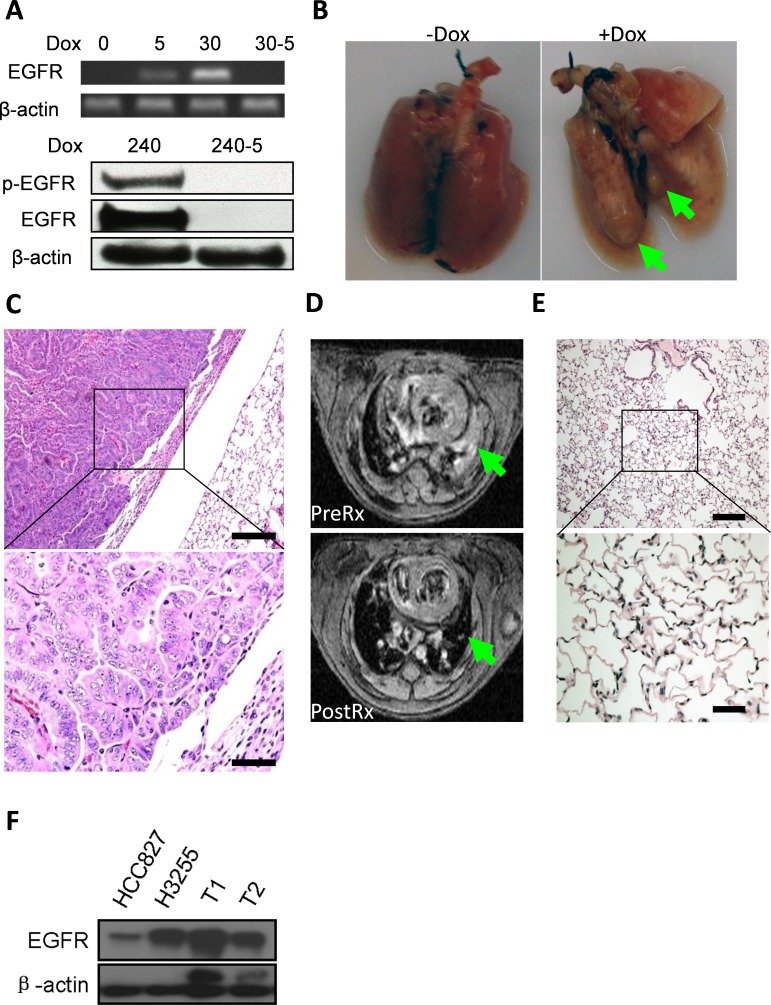
overexpression of wt-EGFR in lung epithelium is tumorigenic and necessary for tumor maintenance *in vivo* **A**. Wt-EGFR expression is tightly controlled in the transgenic mice. Upper panel for mRNA expression in the lung. Lower panel for protein expression in lung tissue before and 5 days after doxycycline withdrawal. All the gels were run under the same experimental conditions. Days for on doxycycline treatment are indicated by number. Withdrawal of doxycycline for 5 days is indicated by “-5”. **B**. Tumor nodules formed in lungs from CC10rtTA/TetO-wtEGFR bitransgenic mice. Left panel, mice not fed with doxycycline. Right panel, mice fed with doxycycline for around 8 months. Large tumor nodules highlighted with arrow-head. **C**. Tumors formed in CC10rtTA/TetO-wtEGFR bitransgenic mice are moderately to poorly differentiated lung adenocarcinoma. **D**. Tumors formed in CC10rtTA/TetO-wtEGFR bitransgenic mice depend on continuous expression of wt-EGFR for maintenance. upper panel: MRI images of mice after 8 months doxycycline induction (PreRx); lower panel: MRI images of same mice after doxycycline withdrawal (PostRx). Tumor region highlighted with green arrow-head. **E**. Histology of the lung of mice withdrawn doxycycline for 2 weeks. Histology (H&E staining) showed nearly normal lung, with occasional area of fibrosis. (scale bar for high magnification 50μm; scale bar for low magnification 200μm). **F**. TetO-wt-EGFR tumors express high amount of EGFR. Tumors from different mice (T1 & T2) were compared against HCC827 (EGFR exon19 deletion) and H3255 (EGFR L858R). Result showed mice tumors express high amount of EGFR.

We next asked whether wt-EGFR overexpression was tumorigenic *in vivo*. We took advantage of the Tet-On system to generate a lung epithelium-specific mouse model for inducible expression of human wt-EGFR. CC10rtTA driver line used in our current research was established by Dr. Jeffery Whitsett and was found capable of driving TetO controlled transgene specifically in the developing and mature respiratory epithelium [33]. We have previously shown that this model is capable of tight control of robust transgenic expression and is capable of inducing lung cancers in mice [34, 35]. 3 transgenic founder lines were found to be capable of developing lung cancers (please see materials and methods). We did all our studies on founder line WC1213 unless otherwise stated (This founder line was found to harbor 3 copies of TetO- wtEGFR transgene, see supplementary Figure 1). RT-PCR analyses detected no mRNA expression of EGFR in CC10rtTA/TetO-wt-EGFR bitransgenic mice and clear expression of EGFR in lung tissues from mice administered doxycycline diet for five days (upper panel, Figure 3A). Longer time (30 days) treatment with doxycycline diet resulted in stronger expression of EGFR. Withdrawal of doxycycline for just five days (30-5) abolished EGFR expression (upper panel, Figure 3A), consistent with tight control of expression we saw in our earlier studies. We also checked EGFR protein level. Strikingly, western blot revealed strong EGFR protein level in mice fed with doxycycline diet for 240 days and EGFR is phosphorylated. Withdrawal of doxycycline diet for just 5 days in these long term doxycycline treated mice resulted in complete regression of EGFR expression (lower panel, Figure 3A).

Twenty-five out of 100 bitransgenic mice exhibit panting behavior after 8 months doxycycline treatment, with a penetrance of around 25%. Large tumor nodules were clearly visible in the lungs, in contrast to normal lungs from control mice (Figure 3B, tumors highlighted with green arrow-head). H&E examination showed that the lung tumors were moderately-to-poorly differentiated lung adenocarcinomas (Figure 3C). To find out whether human wt-EGFR transgene is mutated in the tumor, we isolated genomic DNA from mouse lung tumor nodules and checked mutational status in EGFR transgene through SURVEYOR mutation detection method [36]. Result showed that no mutation existed in the EGFR transgene (data not shown). This solidly showed that wt-EGFR overexpression is tumorigenic *in vivo*.

Oncogene addiction is observed in many tumor models and has been successfully proven in targeting therapies. We next tested whether these lung tumors were addicted to overexpression of wt-EGFR. Tumor burdens were documented by MRI in 2 tumor-bearing mice and these mice were then fed a normal diet without doxycycline for two weeks. Strikingly, we found that tumors completely regressed (data for one representative mouse shown in Figure 3D, upper panel for mouse pre-treatment, lower panel for mouse post-treatment, tumor regions highlighted with green arrow-head. Supplementary Figure 2 for MRI imaging of the other mouse). Histology examination showed that the lungs were almost normal with occasional areas with thickened alveolar walls, indicating remodeling of area originally occupied by tumors (Figure 3E).

To find out the relative EGFR expression level in tumors of our wt-EGFR mice, we harvested lung tissues from our mice, and probed EGFR expression of these tumors against other tissues. Since we are limited by availability of fresh lung cancer samples or snap-frozen lung cancer tissues from patients for this purpose, we compared EGFR expression level in tumors of our mice against lung cancer cell lines known to express high level of EGFR (HCC827 (EGFR exon19 deletion) and H3255 (EGFR L858R)). Our result showed that tumor in wt-EGFR mice expressed equivalent or higher amount of EGFR than H3255 cell line, and much higher amount than HCC827 (Figure 3F).

### wt-EGFR driven lung cancers are sensitive to EGFR targeting reagents

**Figure 4:**
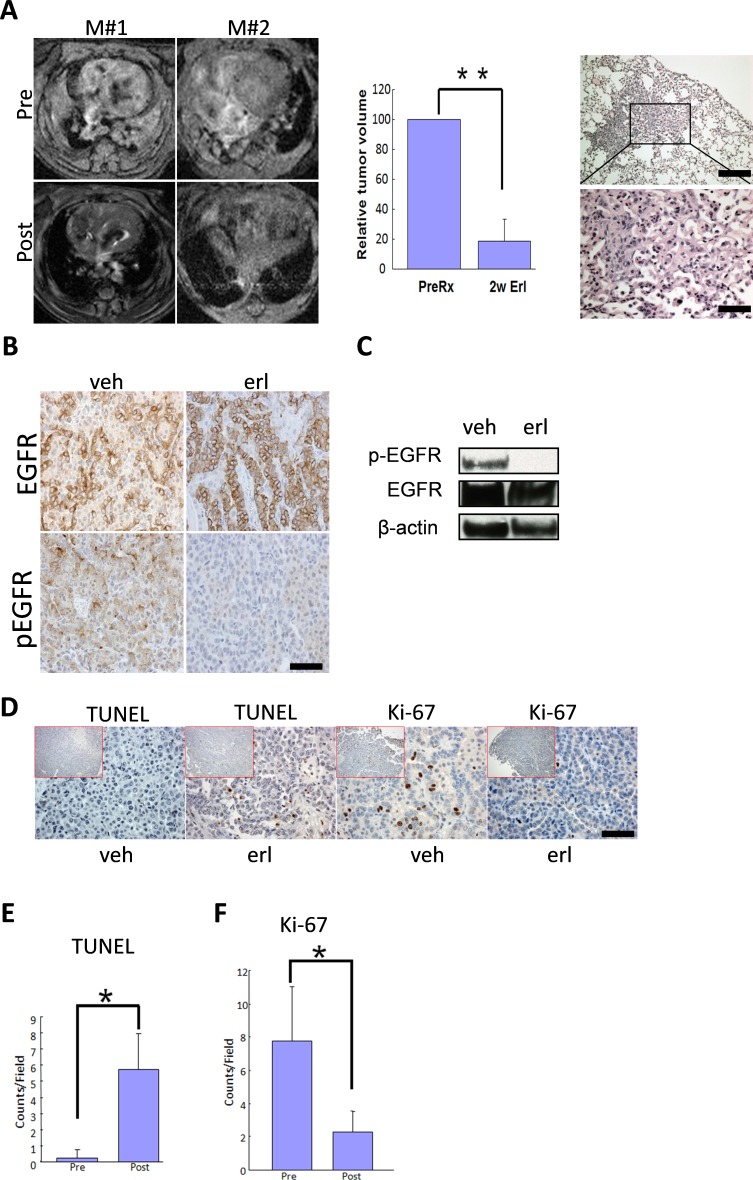
Lung cancers driven by wt-EGFR overexpression are sensitive to erlotinib treatment **A**. Tumor regression in CC10rtTA/TetO-wtEGFR mice in response to erlotinib treatment. Left panel: MRI images showing tumor regression of 2 representative mice (M#1 and M#2), Pre for before erlotinib treatment and Post for after erlotinib treatment; middle panel: quantification of tumor regression on 4 erlotinib treated mice; right panel. Histology of the lung in CC10rtTA/TetO-wtEGFR mice treated for 2 weeks. Lungs are nearly normal. Occasional areas of fibrosis are shown. **B**. wt-EGFR phosphorylation is sensitive to erlotinib treatment for 48 hours. IHC analysis of the lung tumors of mice treated with vehicle and erlotinib is shown. **C**. wt-EGFR phosphorylation is sensitive to erlotinib treatment for 48 hours. Western analysis of the lungs of mice treated with vehicle and erlotinib is shown. All the gels were run under the same experimental conditions. **D**. Erlotinib treatment resulted in arrest of proliferation and promotion of apoptosis in tumors. IHC staining shown for Ki-67 and TUNEL in the tumors from mice treated with vehicle and erlotinib. (scale bar 50 μm) **E**. Statistics of TUNEL staining. **F**. Statistics of Ki-67 staining.

Our transgenic mouse model data confirmed that wt-EGFR-driven lung cancers were dependent on continuous expression of wt-EGFR for maintenance. Moreover, EGFR in these tumors was phosphorylated. We therefore tested whether these tumors were sensitive to EGFR inhibition with TKIs. We fed a cohort of mice with doxycycline diet for around 8 months and tumor burdens were documented with MRI in 4 mice before and after treatment with erlotinib for 2 weeks. Data showed that these tumors were highly sensitive, with complete regression after two weeks of erlotinib treatment in all of these 4 mice. Histology examination revealed largely normal lungs with occasional areas of heavy fibrosis, suggesting remodeling of lung regions originally occupied by tumors (Figure 4A, left panel for MRI images of 2 representative mice; middle panel for quantification of tumor regression; and right panel for histology examination; supplementary Figure 3 for the MRI imaging data for all 4 mice).

To verify EGFR inhibition by erlotinib in these tumors, we treated another cohort of mice with vehicle or erlotinib daily for two days and sacrificed these mice 4 h after last dosing. Immunohistochemical staining revealed that EGFR was strongly expressed and phosphorylated in these tumors, suggesting that EGFR was activated in these tumors. After short-term treatment with EGFR TKIs, we found that EGFR expression was unaffected, but that EGFR was dephosphorylated in tumors (Figure 4B). We further verified this conclusion by Western blot analysis on isolated tumor nodules (Figure 4C).

We next examined the impact of EGFR TKIs on tumor cell proliferation and apoptosis through immunohistochemistry. TUNEL staining (apoptotic signal) detected very few positive tumor cells in the vehicle treated mice, suggesting of very few apoptotic tumor cells. In striking contrast, tumors are strongly positive for TUNEL signals 48 h after erlotinib treatment (Figure 4D). Ki-67 staining detected a significant portion of positive tumor cell in vehicle treated mice, suggesting that tumor cells were proliferating quickly. Erlotinib treatment resulted in a significant decrease of Ki-67 signals in tumor (Figure 4D, Statistics in Figure 4E for TUNEL and Figure 4F for Ki-67). This results showed that erlotinib treatment led the tumor cells to exit of cell cycle and elicit apoptosis in these tumor cells.

In addition to small molecules, antibodies are another category capable of inhibiting EGFR kinase activity. Cetuximab, through binding to the extracellular domain of EGFR to prevent activation of intracellular kinase domain, is now a clinically available EGFR inhibitor [37]. We also checked the efficacy of cetuximab against wt-EGFR driven lung cancer in our mouse model. Our data showed that these tumors respond significantly to cetuximab treatment in all of the 3 mice tested (supplementary Figure 4).

### EGFR expression level correlated with patients’ longer overall survival after TKI treatment

Our clinical data have shown that a small portion of NSCLC patients negative of EGFR mutations respond to TKI treatment as judged by CT images. OS is the major primary endpoint for assaying TKI benefit. We therefore went on to check whether IHC score of EGFR staining in mutation-negative patients are correlated with overall survival after TKI treatment.

**Figure 5:**
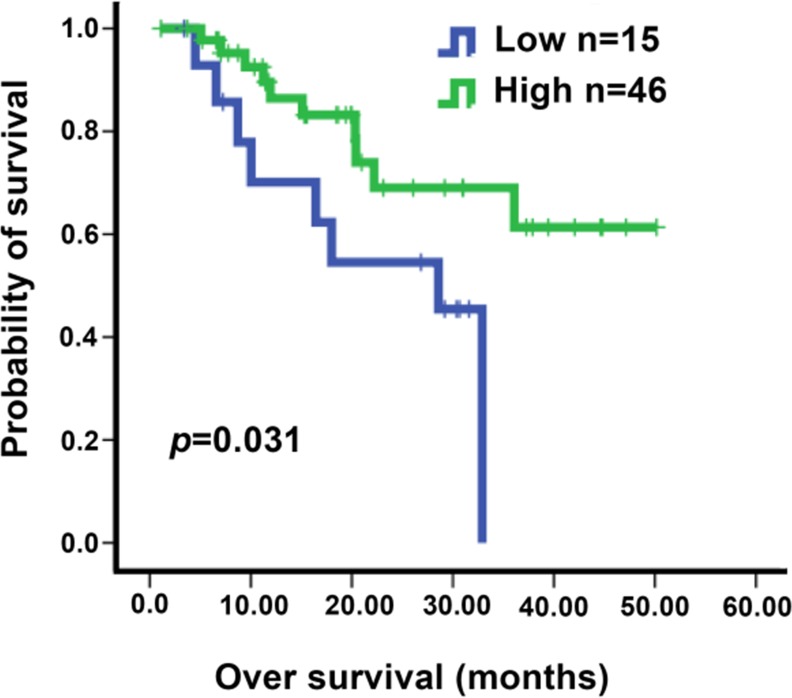
Kaplan-Meier analysis of overall survival according to EGFR IHC score in NSCLC patients Patients were all treated with gefitinib. The *H* scores of 100 was chosen as the cutoff point for separating tumors with high or low EGFR expression. The *P* value for the difference between the two curves was determined by the log-rank test (*P* = 0.031).

We screened 3543 NSCLC patients treated with EGFR TKI at our hospital (Sun Yat-Sen University Cancer Center) during January 2008 to March 2014 for negativity of EGFR mutation, record of detailed medical history and availability of enough tumor tissue samples for IHC analysis. 61 patients harboring advanced lung cancers treated with gefitinib qualified for further analysis (please see Supplementary Table 3 for the detailed information of the patients). We notice that around 9.8% of wt-EGFR patients showing partial regression of lung cancer, and another 52% stable disease (Supplementary Table 3). Baseline demographic characteristics, multivariate analyses of PFS and OS, and duration of response types among patients are listed in Supplementary Tables 4, 5, and 6. IHC was conducted to assay for EGFR expression level in tumors and identified 46 patients harboring tumors expressing high level of EGFR and 15 low-EGFR patients. Interestingly, we found that higher expression level is correlated with significantly longer patients’ survival (Figure 5).

## DISCUSSION

In our current work, we have shown that wt-EGFR-overexpressing patients respond to TKI treatment. We have also shown that overexpression of wt-EGFR transforms cells *in vitro* and is tumorigenic *in vivo*. Using mice transgenic for lung epithelium-specific expression of human wt-EGFR, we have shown that tumors driven by overexpression of wt-EGFR are highly sensitive to TKIs. NSCLC patients overexpressing wt-EGFR benefit from TKI treatment by showing longer OS. Our work thus shows that a clinical trial is warranted to determine efficacy of targeting therapy for wt-EGFR overexperssing lung cancer patients.

Earlier reports hinted that overexpression of wt-EGFR transforms cells [38-40]. Zhang *et al*. showed that wt-EGFR tends to be activated at high local concentrations [28]. Moreover, EGFR ligands are detectable in physiolological conditions. Wt-EGFR overexpressing 3T3 cells is capable of forming tumor nodules in nude mice [38]. These studies, together with our transgenic mouse model that overexpression of wt-EGFR in lung epithelium leads to lung cancer development make a strong argument for tumorigenicity of overexpression of wt-EGFR in patients. High level of wt-EGFR protein in the patients’ lung epithelium can be activated by at least the following two means: high local concentration may lead to activation of EGFR; ligands in serum can stimulate wt-EGFR to activate its signal transduction.

EGFR expression and function is controlled by many mechanisms. Promoter polymorphism at −216 and −191 impact protein expression [41, 42]. Polysomy or copy number gain is detected in some patients. Also, autocrine or paracrine ligand-to-receptor loops could potentially activate EGFR, as co-expression of EGFR and TGFa was detected in the same tumor [43]. EGFR is also negatively controlled. CBL E3 ligase targets the EGFR protein for degradation [44]. Phosphatases dephosphorylate EGFR and thus quench EGFR signaling [45].

Earlier retrospective studies hinted TKI benefits in EGFR mutation negative patients [46, 47]. Our current work clearly showed that TKI treatment benefits the wt-EGFR overexpressing patients. Considering those reports that EGFR copy number, protein level, phospho-EGFR level were correlated with benefits of TKI treatment in patients, we believe that generally EGFR function plays a critical role in tumor maintenance in a portion of patients and that these patients can benefit from TKI treatment. In those cases that discrepancy were reported, negative regulators could possibly explain inconsistency.

Currently, clinics follow ASCO 2011 and NCCN 2013 guidelines that demand that lung cancers be genotyped to confirm EGFR mutations before administering TKIs. These guidelines exclude wt-EGFR lung cancer patients for TKI administration. However, we show here that there exists a portion of patients who is negative for EGFR mutation but benefits from TKI treatment. Thus, a clinical trial is warranted to determine the biomarkers for selecting patients and efficacy of EGFR TKIs in this mutation negative population.

## MATERIALS AND METHODS

### Mouse experiment

Transgenic TetO-human wt-EGFR mice were constructed as we reported earlier [34]. 9 founder lines were crossed with CCSP-rtTA mice (generously provided by Dr. Jeffery Whitsett). Bitransgenic offspring of three founders (WC1213, WC1246, WC1247) were identified as capable of developing lung tumors after putting on doxycycline. All mice were housed in a pathogen-free environment at the National Institute of Biological Sciences, Beijing (NIBS). All experimental protocols were approved by the Institutional Committee for Animal Care and Use, NIBS. Animal work was carried out in good accordance to the approved protocol. Erlotinib treatment was carried out as reported in our earlier work [34]. Magnetic resonance imaging of mouse lung tumor was conducted as reported [34].

### Ethics statement

The protocol was approved by Sun Yat-Sen University Cancer Center, Guangdong, China, and the Institutional Committee at the National Institute of Biological Sciences, Beijing (NIBS). Written consent was obtained from every patient who donated tissues. All work was carried out in good accordance to the approved protocol.

### Cell culture

BEAS-2B and NIH-3T3 cells were purchased from ATCC. BEAS-2B cells were transfected with linearized CAG-rtTA-IN using Lipofectamine 2000 (Invitrogen) and infected with pREV-TRE-wt-EGFR retrovirus. Cells were selected with G418 (700 μg/mL) and hygromycin (200 μg/mL) to produce low EGFR-expressing wt-EGFR tet-on BEAS-2B cells as these cells exhibit low level of background leakiness of wt-EGFR expression in the absence of doxycycline. BEAS-2B cells were transfected with linearized CAG-wt-EGFR-IN and selected with G418 (700 μg/mL) to produce high-expressing cells. NIH-3T3 cells were transfected with linearized CAG-wt-EGFR-IN or Rosa-wt-EGFR-IN and selected with G418 (500 μg/mL) to produce high- or low-expressing cells, respectively. BEAS-2B cells were cultured in DMEM supplemented with 10% FBS (Gibco). NIH-3T3 cells were cultured in DMEM containing 10% FBS (Hyclone). All cell culture media were supplemented with 10 mM glutamine and 1% penicillin and streptomycin and incubated at 37°C in a humidified incubator with 5% CO_2_. All plasmids and related information are available upon request.

### Soft agar assay

BEAS-2B cells with high- or low-expressing wt-EGFR were seeded in soft agar in 6-well plates (10,000 cells per well). Top agar contained 0.35% low melting point agarose (Invitrogen) with DMEM containing 10% FBS (Gibco). Base agar consisted of DMEM with 0.5% high melting point agarose (Invitrogen) and 10% FBS. In EGF-positive assays, both layers contained 20 ng/mL EGF (R&D system). Colonies were counted two weeks after seeding in soft agar. Assays were carried out in triplicate for quantification.

NIH-3T3 high- or low-expressing cells were seeded in soft agar in 6-well plates (100,000 cells per well). Top agar contained 0.35% low melting point agarose (Invitrogen) with 10% FBS (Hyclone) and DMEM. Base agar consisted of DMEM with 0.5% high melting point agarose (Invitrogen) and 10% FBS. In EGF-positive assays, both layers contained 20 ng/mL EGF (donated by Beigene Co.). Colonies were counted twenty days after seeding in soft agar. Assays were carried out in triplicate for quantification.

### Western blot analysis

All BEAS-2B and NIH-3T3 cells were deprived of serum overnight. Before EGF stimulation, starved cells were treated with the indicated amount of erlotinib (donated by Xiaoguang Lei) for 4 h. After EGF stimulation (20 ng/mL) for 20 min, cells were lysed in RIPA buffer (Beyotime) supplemented with protease and phosphatase inhibitors (Roche). Western blotting was performed using standard methods. Blots were probed with the following antibodies: β-actin (Sigma), EGFR, phospho-EGFR (p1068 and p1173), AKT, phospho-AKT-Ser473 (pAKT), ERK1/2, phospho-ERK1/2 -Thr202/Tyr204 (Cell Signaling Technology).

### H&E staining and IHC

The formalin fixed lung tissues from mice or patients were embedded in paraffin and 5-μm-thick slices were sectioned. H&E and IHC staining were performed using standard methods to detect total EGFR and phospho-EGFR.

### Statistical analysis

All quantified results were analyzed by GraphPad Prism. Two-tailed paired *t* tests were performed to evaluate the significance. For bar graphs, data are represented as mean ±SEM. The *P* values were denoted as * (*p* < 0.05), ** (*p* < 0.01), and *** (*p* < 0.001).

### Patients survival analysis

3543 patients diagnosed with NSCLC and treated with EGFR-TKIs at Sun Yat-Sen University Cancer Center (Guangzhou, China) during January 2008 to March 2014 were checked in the study. 61 patients harboring advanced lung cancers were identified with known EGFR mutation negative status, with detailed medical records, and with enough tumor tissue samples available for checking EGFR protein levels through immunohistochemical staining. SPSS 13.0 software was used for the statistical analysis. The cut-off values were obtained by X-tile software (Version 3.6.1, Yale University, New Haven, CT). The H-score of 100 was chosen as the cutoff for grouping tumors into high and low (≤100 was low and > 100 was high, respectively) expression of EGFR. The Kaplan-Meier method was used for analyzing overall survival (OS) and log-rank test was adopted to assess the possible individual risk factors related with survival.

## SUPPLEMENTARY MATERIAL FIGURES AND TABLES






