# Correction: Overexpression of wildtype EGFR is tumorigenic and denotes a therapeutic target in non-small cell lung cancer

**DOI:** 10.18632/oncotarget.26254

**Published:** 2018-10-16

**Authors:** Naiqing Xu, Wenfeng Fang, Libing Mu, Yanna Tang, Lei Gao, Shengxiang Ren, Dengfeng Cao, Lixin Zhou, Aiqun Zhang, Deruo Liu, Caicun Zhou, Kwok-Kin Wong, Lei Yu, Li Zhang, Liang Chen

**Affiliations:** ^1^ Graduate School of Peking Union Medical College, Beijing, China; ^2^ National Institute of Biological Sciences, Beijing, China; ^3^ Chinese Academy of Medical Sciences, Beijing, China; ^4^ Collaborative Innovation Center for Cancer Medicine, Sun Yat-Sen University Cancer Center, Guangzhou, Guangdong, P. R. China; ^5^ Tsinghua University School of Medicine, Beijing, China; ^6^ Shanghai Pulmonary Hospital, Shanghai, China; ^7^ Cancer Hospital and Institute, Chinese Academy of Medical Sciences, Beijing, China; ^8^ Peking University Cancer Hospital, Beijing, China; ^9^ General Hospital of People’s Liberation Army, Beijing, China; ^10^ Department of Thoracic Surgery, China-Japan Friendship Hospital, Beijing, China; ^11^ Department of Medical Oncology, Dana-Farber Cancer Institute, Boston, MA, USA; ^12^ Beijing Tongren Hospital, Capital Medical University, Beijing, China; ^13^ National Institute of Biological Sciences, Collaborative Innovation Center for Cancer Medicine, Beijing, China

**This article has been corrected:** The correct Grant Support information is given below:

**GRANT SUPPORT**

This work was funded by National Natural Science Foundation of China grant 81472606, Chinese Ministry of Science and Technology grant (973 grant) 2011CB812401, and the Beijing Municipal Government.

Original article: Oncotarget. 2016; 7:3884-3896. https://doi.org/10.18632/oncotarget.6461

