# Supplementary material for: Overexpression of wildtype EGFR is tumorigenic and denotes a therapeutic target in non-small cell lung cancer
Source: Oncotarget. 2015 Dec 4;7(4):3884–96. doi: 10.18632/oncotarget.6461 (PMC4826177; doi:10.18632/oncotarget.6461)
Supplement: Supplementary file 1 [file oncotarget-07-3884-s001.pdf]

Overexpression of wildtype EGFR is tumorigenic and denotes a therapeutic target in non-small cell lung cancer

Supplementary Material

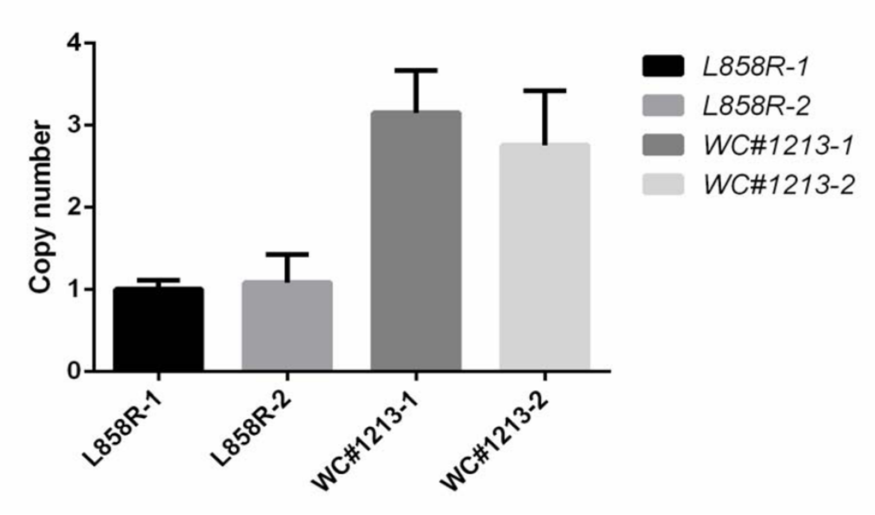

Supplementary figure 1

Founder line WC1213 harbors 3 copies of TetOnwtEGFR transgene

Tail DNA was isolated from our earlier established transgenic EGFR L858R mice (singlecopy) and WC1213 transgenic mice. Realtime PCR was carried out with primers indicated below. 2 mice each was used. Result indicated that WC1213 founder line harbored 3 copies of transgene. EGFRF ATCCAGTGTGCCCACTACATTG EGFRR TACTTCCAGACCAGGGTGTTGT ActinF acagcttctctttgatgtcacgc ActinR tgtgatggactccggagacgg

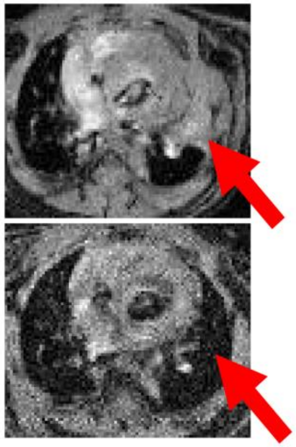

Supplementary figure 2

wtEGFR driven lung cancers depend on continuous expression of EGFR transgene for maintenance

CC10rtTA/Tet-OnEGFR bitransgenic mice were fed with doxycycline diet for around 8 months and then subjected to doxycycline withdrawal for 2 weeks. MRI images recorded before (upper panel) and after (lower panel) doxycycline withdrawal showed complete tumor regression. Tumor region highlighted with red arrow head.

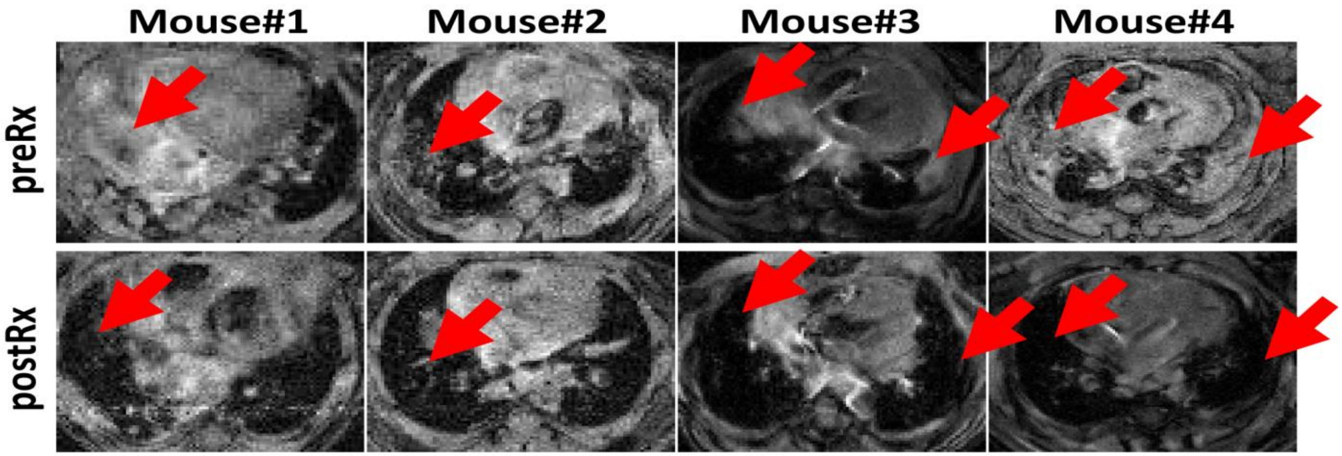

Supplementary figure 3

Wt EGFR driven lung cancers are sensitive to erlotinib treatment CC10rtTA/TetO wt EGFR bitransgenic mice were fed with doxycycline diet for around 8 months and then treated with erlotinib for 2 weeks. MRI images recorded before (upper panel) and after (lower panel) erlotinib treatment showed dramatic tumor regression. Tumor region highlighted with red arrow head. Result revealed tumor regression in all 4 mice

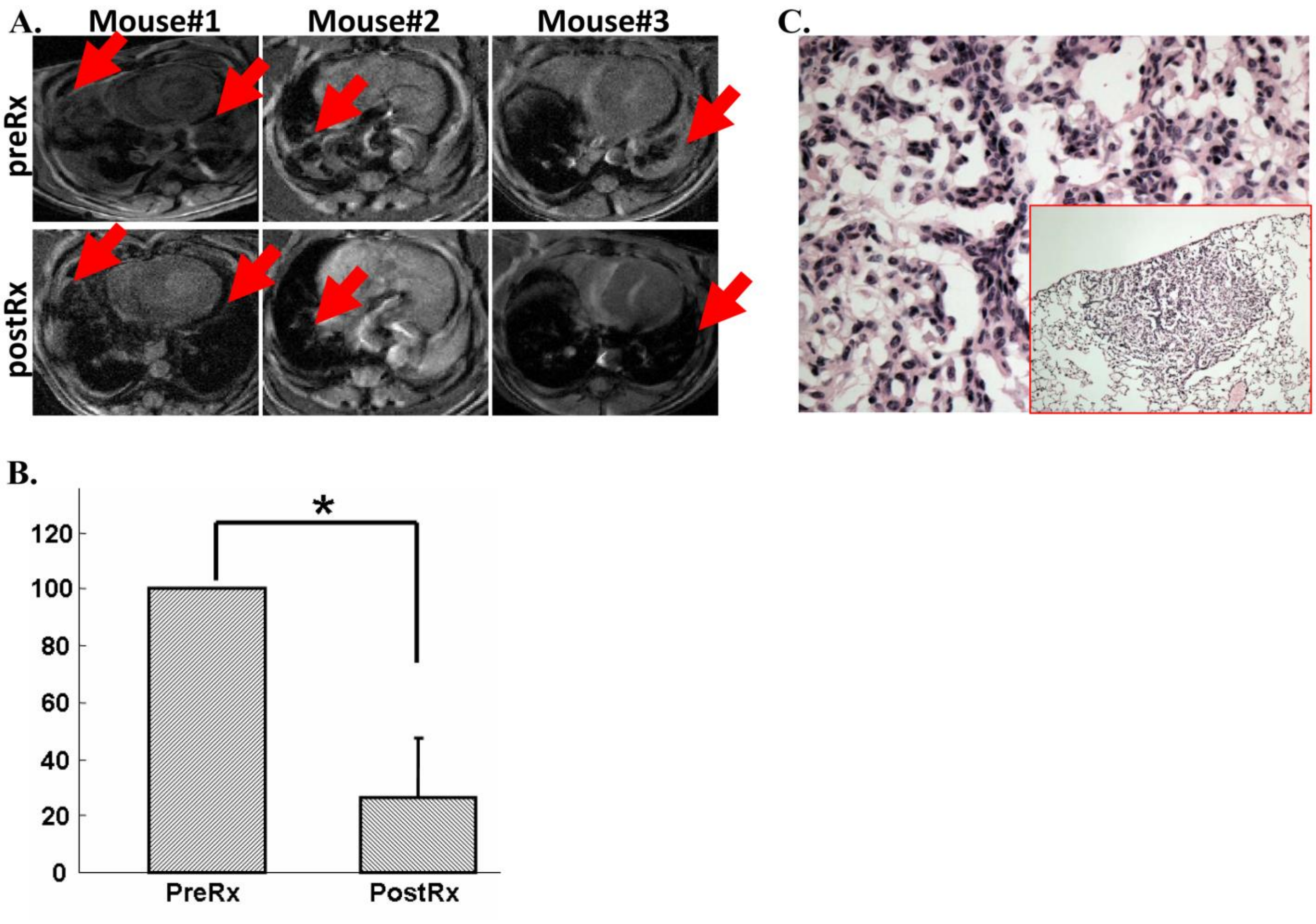

Supplementary figure 4

Wt EGFR driven lung cancers are sensitive to cetuximab treatment

Three CC10rtTA/TetO wt EGFR bitransgenic mice were fed with doxycycline diet for around 8 months and then treated with cetuximab for 2 weeks. **A.** MRI images recorded before (upper panel) and after (lower panel) cetuximab treatment showed dramatic tumor regression. Tumor region highlighted with red arrow head. **B.** Quantification of tumor regression.

C. H&E analysis of the lung pathology revealed almost more histology with occasional areas of fibrosis indicating remodeling of original tumor area.

Supplementary table 1: OncoCarta™ Panel v1.0—mutations list

（total 19 oncogenes and 238 sites）

**oncogene（mutational sites）**	**Chromosomal locus**	**Mutational detail**	
ABL1(14)	9q34.1	G250E, Q252H, Y253H, Y253F, E255K, E255V,D276G, F311L, T315I, F317L, M351T, E355G,F359V, H396R	
AKT1(7)	14q32.32	V461L, P388T, L357P, E319G, V167A, Q43X, E17K	
AKT2(2)	9q13.1-q13.2	S302G, R371H	
BRAF(24)	7q34	G464R, G464V/E, G466R, F468C, G469S, G469E,G469A, G469V, G469R, G469R, D594V/G, F595L,G596R, L597S, L597R, L597Q, L597V, T599I,V600E, V600K, V600R, V600L, K601N, K601E	
CDK(2)	12q14	R24C, R24H	

EGFR(43)	7p21	R108K, T263P, A289V, G598V, E709K/H, E709A/G/V, G719S/C, G719A, M766_A767insAI, S768I, V769_D770insASV, V769_D770insCV, D770_N771>AGG/N771>GF/N771T, D770_N771insG, V769_D770insASV, D770_N771insGF/D770_N771insGL, D770fs*61/D770_N771insAPW, P772_H773insV, H773N, H773_V774insNPH/PH/H, V774_C775insHV, T790M, L858R, L861Q, E746_A750del, E746_T751del, E746_T751>A/V/S, E746_S752>V/A/D/I, L747_E749del, L747S, L747_A750del, L747_A750>P, L747_T751del, L747_T751>P, L747_S752del, L747_P753>S/Q, A750P, T751A, T751P, T751I, S752F, S752_I759del, P753Q,	
ERBB2(7)	17q21.1	L755P, G776S/LC, G776VC/VC,A775_G776insYVMA, P780_Y781insGSP,P780_Y781insGSP, S779_P780insVGS	
FGFR1(2)	8p11.2-p11.1	S125L, P252T	
FGFR3(5)	4p16.3	G370C, Y373C, A391E, K650Q/E, K650T/M	
FLT3(3)	13q12	I836del, D835H/Y	
HRAS(6)	11p15.5	G12V/D, G13C/R/S, Q61H/H, Q61L/R/P, Q61K	
JAK(1)	9p24	V617F	
KIT(27)	4q11-q12	D52N, Y503_F504insAY, W557R/R/G, V559D/A/G,V559I, V560D/G, K550_K558del, K558_V560del,K558_E562del, V559del, V559_V560del, V560del,Y570_L576del, E561K, L576P, P585P, D579del,K642E, D816V, D816H/Y, V825A, E839K, M552L,Y568D, F584S, P551_V555del, Y553_Q556del	

KRAS(12)	12p12.1	G12C, G12R, G12S, G12V, G12D, G12A, G12F,G13V/D, A59T, Q61E/K, Q61L/R/P, Q61H/H	
MET(5)	7q31	R970C, T992I, Y1230C, Y1235D, M1250T	
NRAS(8)	1p13.2	G12V/A/D, G12C/R/S, G13V/A/D, G13C/R/S, A18T,Q61L/R/P, Q61H, Q61E/K	
PDGFRA(11)	7p22	V561D, T674I, F808L, D846Y, N870S, D1071N,D842_H845del, I843_D846del, S566_E571>K,I843_S847>T, D842V	
PIK3CA(13)	3q26.3	R88Q, N345K, C420R, P539R, E542K, E545K,Q546K, H701P, H1047R/L, H1047Y, R38H, C901F,M1043I	
RET(6)	10q11.2	C634R, C634W, C634Y, E632_L633del, M918T,A664D	

Supplementary Table 4 Clinicopathological characteristics of 61 NSCLC patients with wild-type EGFR

**Age at diagnosis, y**	
mean (SD)	55.8±9.5
median	55.0
range	36.0-77.0
**Sex, No. (%)**	
women	18 (29.5)
men	43 (70.5)
**Pathology, No. (%)**	
ADC	48 (78.7)
non-ADC	13 (21.3)
**Stage, No. (%)**	
ⅢB	1 (1.6)
Ⅳ	60 (98.4)
**Gefitinib, No. (%)**	
1st line	19 (31.1)
≥2nd line	42 (68.9)
**EGFR h-score, No. (%)**	
EGFR positive	31 (50.8)

EGFR negative	30 (49.2)
**Gefitinib’s best response, No. (%)**	
PR	6(9.8%)
SD	32(52.5%)
PD	23(37.7%)

Abbreviations: PD-L1, programmed cell death-ligand 1; ADC, adenocarcinoma; non-ADC, non-adenocarcinoma; EGFR, epidermal growth factor receptor; EGFR-TKI, EGFR-tyrosine kinase inhibitor.

Supplementary Table 5 Multivariate analyses of PFS and OS

Parameter	PFS Multivariate analysis					OS Multivariate analysis				
		N	HR	95%CI	P-value		N	HR	95%CI	P-value
**Age**										
≤50y	1	17				1	17			
>50y		44	1.000	0.439-2.280	1.000		44	2.250	0.553-9.153	0.257
**Sex**										
female	1	18				1	18			
male		43	1.000	0.489-2.045	1.000		43	1.185	0.395-3.555	0.762
**Pathology**										
ADC	1	48				1	48			
non-ADC		13	1.000	0.366-2.734	1.000		13	1.316	0.306-5.567	0.712
**Stage**										
IIIB	1	1				1	1			
IV		60	1.000	0.000-5118736	0.750		60	0.159	0.016-1.563	0.115

**Gefitinib**										
1st line	1	19				1	19			
≥2nd line		42	1.000	0.509-1.963	1.000		42	0.963	0.358-2.589	0.941
**EGFR h-score**										
negative	1	30				1	30			
positive		31	1.000	0.513-1.950	1.000		31	0.606	0.220-1.669	0.332

Abbreviations: OS, overall survival; PFS, progression-free survival; HR, hazard ratio; 95%CI, 95% confidence intervals; ADC, adenocarcinoma; non-ADC, non-adenocarcinoma; EGFR, epidermal growth factor receptor; EGFR-TKI, EGFR-tyrosine kinase inhibitor.

Supplementary Table 6 duration of response types among patients

Response type	N	PFS (months, mean±SD)
PR	6	12.77±10.72
SD	32	7.43±8.80
PD	23	1.32±1.23
